# Snakes of Ko Pha-ngan in the Surat Thani Province, Thailand

**DOI:** 10.3897/BDJ.14.e199297

**Published:** 2026-09-10

**Authors:** Dawn R. Renae Cook-Price, Sunchai Makchai, Pongthep Suwanwaree

**Affiliations:** 1 Suranaree University of Technology, Nakhon Ratchasima, Thailand Suranaree University of Technology Nakhon Ratchasima Thailand; 2 Thailand Natural History Museum, Pathum Thani, Thailand Thailand Natural History Museum Pathum Thani Thailand

**Keywords:** biodiversity, conservation, insular populations, island biogeography, species list, reptiles

## Abstract

**Background:**

Insular snake species are often under-assessed, rendering them vulnerable to habitat encroachment and other anthropogenic threats. The aim of this study was to compile a comprehensive checklist of snakes on Ko Pha-ngan, Thailand. Data were collected via transect surveys, drift line fence traps and opportunistic encounters from February 2021 to September 2023.

**New information:**

Twenty-one snake species were detected across three main habitats (human settlement, human-disturbed forest and national park forest) with the highest species diversity observed in the Than Sadet - Ko Pha-ngan National Park protected areas (20 species). *Lycodon capucinus*, *Chrysopelea ornata*, *Ahaetulla prasina* and *Boiga cyanea* were the most abundant species detected during surveys. Thirteen species were detected in all three habitat types, indicating broad habitat tolerance amongst several taxa. Two species, *Ptyas korros* and *Ophiophagus bungarus*, are assessed as Near Threatened and Vulnerable, respectively, according to the IUCN Red List of Threatened Species. Finally, the island harbours one endemic species, *Oligodon phangan*. This study highlights the need for additional monitoring to better understand the dynamics of insular populations and to assess the ongoing impacts of tourism-driven development on species within finite island habitats.

## Introduction

Earth contains remarkable biodiversity, approximately 20% of which occurs on islands (Fernández-Palacios et al. 2021). Insular ecosystems are often celebrated for their rich biodiversity, but remain particularly vulnerable to human-induced change, disproportionately housing approximately half the world's threatened species (Russell and Kueffer 2019, Fernández-Palacios et al. 2021). Across tropical islands, rapid tourism-driven development, agricultural expansion and deforestation have altered habitats, reduced ecological connectivity and increased human-wildlife conflict, placing pressure on even the most resilient vertebrate groups (Cox et al. 2022).

Amongst vertebrates, snakes represent a vital, yet often overlooked component of ecosystems (Lillywhite and Martins 2019). As mesopredators, they regulate rodent and amphibian populations, contribute to seed dispersal as secondary dispersers and maintain trophic balance (Beri and Bhaumik 2021). However, their secretive nature, combined with persistent cultural fear and persecution, means they are disproportionately affected by habitat fragmentation, edge effects and negative public perception (Kjoss and Litvaitis 2001). Despite their ecological importance, snakes remain one of the least studied vertebrate groups with only 20% of insular species surveyed in the past decade (Bonnet et al. 2002). A recent review of snake venom research found that viperids were significantly over-represented in literature and that most studies focused on venom, whereas ecological and functional investigations were comparatively rare (Avella et al. 2022). Unfortunately, this means fundamental information regarding species occurrence and community composition remains lacking for many island systems in South East Asia.

Long-standing myths and misconceptions about snakes have further complicated their conservation, making the study of their ecological roles both timely and crucial (Mullin and Seigel 2011). Far from these villainising misunderstandings, snakes provide valuable ecosystem services that shape vertebrate and invertebrate communities through trophic cascades (Beri and Bhaumik 2021).

During the late Pleistocene, when sea levels were lower, Ko Pha-ngan formed part of the Sunda Shelf; subsequent Holocene sea-level rise isolated the island (Nutalaya et al. 1979, Sathiamurthy and Voris 2006). At a deeper timescale, the island sits on the Sibumasu (Shan-Thai) Terrane, a peri-Gondwanan fragment forming much of Peninsular Thailand and Malaysia (Cocks and Torsvik 2002,Cocks et al. 2005). The peninsula’s spine is the late Triassic-early Jurassic main range Batholith (Hutchison 1996), whose granites underlie the region and adjacent islands. This continental foundation distinguishes Ko Pha-ngan from oceanic islands.

As sea levels rose so too did the island’s isolation which had profound effects on biodiversity. This geological shift, coupled with ongoing anthropogenic pressures, has significantly influenced the island's biodiversity. With the advancement of human technology and increase in human settlements, islands in Southeast Asia, including Ko Pha-ngan, faced significant deforestation (Wood et al. 2017).

Ko Pha-ngan exemplifies the duality of picturesque beaches with vibrant tourism and ever-increasing habitat fragmentation. Historical land-use practices have profoundly shaped the ecological landscape of Ko Pha-ngan. The island experienced extensive tin mining in the 1970s, followed by widespread coconut plantation development, which decimated large tracts of primary forest (Nutalaya et al. 1979). Today, Ko Pha-ngan is a prominent tourist destination, attracting over one million visitors annually, driven largely by its famous Full Moon Party (Kaewcharoen et al. 2019); however, this tourism-driven development has intensified habitat fragmentation, posing significant threats to the survival of many species, particularly snakes. Habitat loss and fragmentation remain amongst the leading drivers of biodiversity decline globally and are especially critical for snake populations, given their reliance on intact ecosystems.

Although Thailand is home to more than 500 reptile species (Poyarkov et al. 2023), research on the snakes of its islands remains sparse. Ko Pha-ngan, in particular, has received limited scientific attention despite its ecological significance. Unlike neighbouring areas, such as the Phi Phi Archipelago or mainland locations like the Bang Saphan District, which have been the focus of herpetological surveys (Milto 2014, Lerdrungroj and Taksintum 2018), Ko Pha-ngan’s snake species remain largely undocumented. Approximately one-third of the island is included within the Than Sadet - Ko Pha-ngan National Park (Department of National Parks, Wildlife and Plant Conservation 2018), yet the degree to which this protected area safeguards snake populations is unknown.

By documenting the diversity of snake species on Ko Pha-ngan, this study aims to establish a critical baseline for future conservation efforts of this island. Snakes play a crucial role in maintaining ecosystem balance as both predators and prey, yet their populations are particularly sensitive to habitat fragmentation. This study seeks to bridge this knowledge gap by establishing a baseline assessment of the current status of snake species on Ko Pha-ngan.

## Materials and methods

Occupying 125 km^2^ (15 km north to south and 10 km east to west) in the Gulf of Thailand, Ko Pha-ngan is located 80 km off the east coast of peninsular Thailand. The island is home to the Than Sadet-Ko Pha-ngan National Park which covers approximately one-third of the island (42.9 km^2^) and a maximum elevation of 635 m for the entire island with over half the island consisting of montane forest. While, in contrast, most lowland areas are used for residential and agriculture purposes (Department of National Parks, Wildlife and Plant Conservation 2020, Koh Phangan City 2023).

From February 2021 through September 2023, surveys were conducted on the island of Pha-ngan approximately twice a week for a total of 78 weeks. A total of 1,343 survey hours were completed between 19:00 and 02:00 h throughout the survey period.

**Figure 1.**
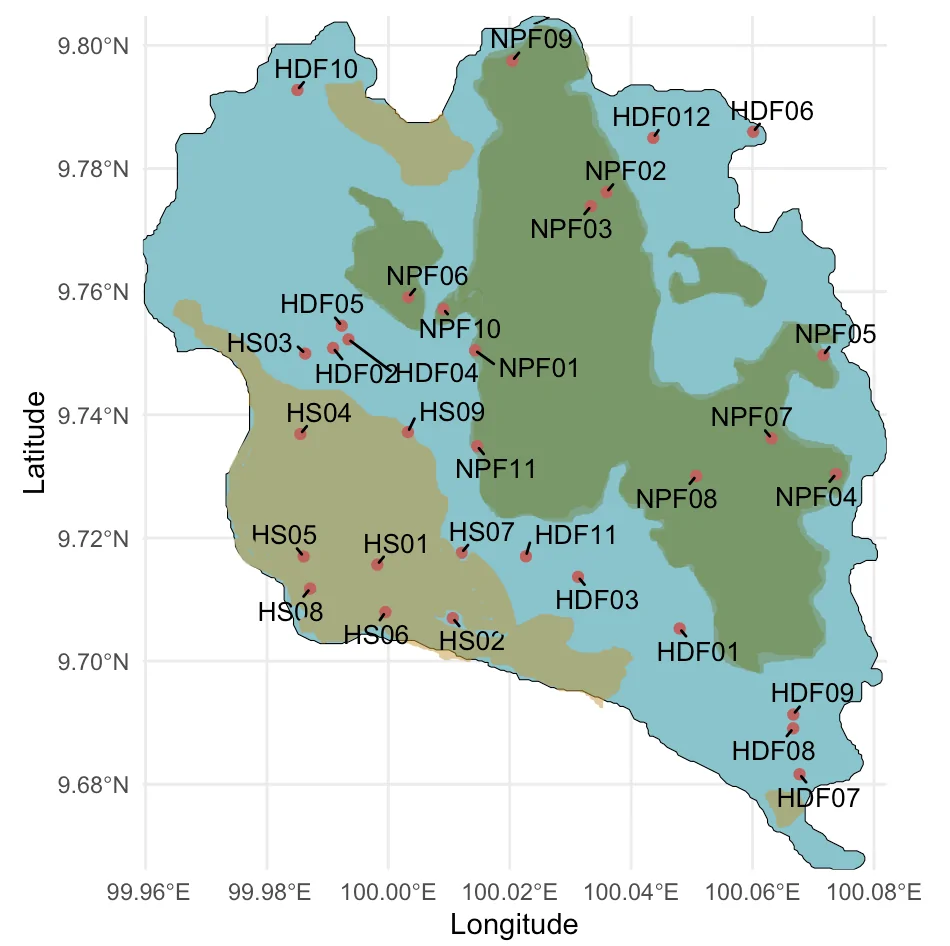
Transect locations and three habitat types of Ko Pha-ngan, Thailand. Human settlement (HS), human-disturbed forest (HDF) and national park forest (NPF).

**Figure 2.**
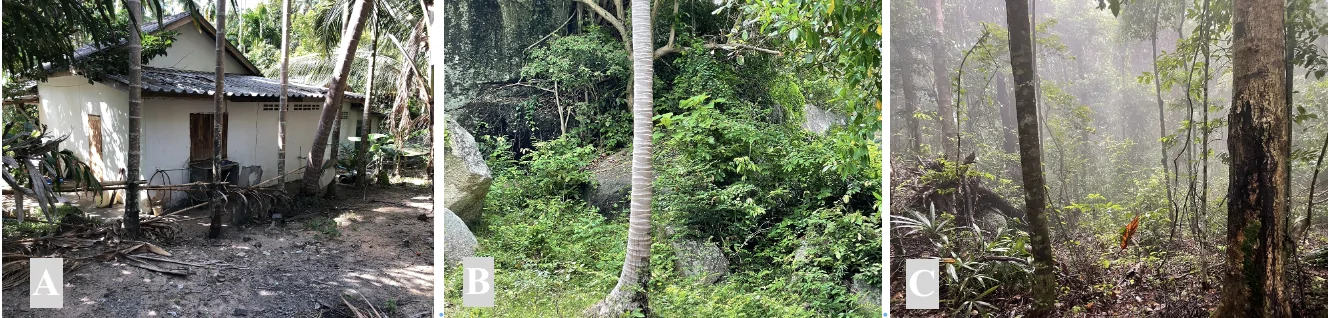
Habitat types. **A** human-settlement; **B** human-disturbed forest; **C** national park forest. All photos are where species have been found.

There were 32 survey transects utilised, spanning three different habitat categories (Figs 1, 2); human settlement (HS), human-disturbed forest (HDF) and national park forest (NPF). Human-disturbed forest is any patch of fragmented forest area near human settlements or witnessing human activity in sections throughout (Cremonesi et al. 2022). Transect lengths were not standardised and varied between 500 m and 1.5 km with variation in elevation from sea level to 630 m. Despite the varying transect distance, only one geographic point was recorded for the survey site.

The purpose of this study was to document the species for the entire island and, therefore, remote areas with varying habitats were sought out. Multiple factors such as proximity to water, access to private land and challenging terrain, were used to determine transect locations. Transect lengths varied, often due to terrain limitations.

**Figure 3.**
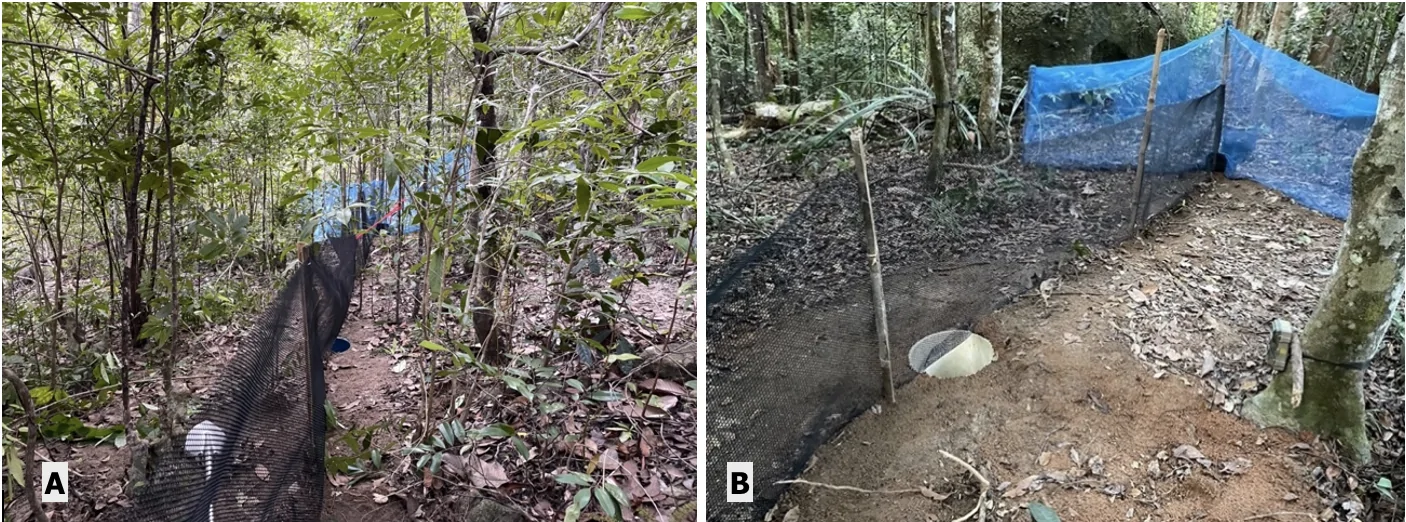
Drift-line fence trap; **A** complete view; **B** funnel trap used in conjunction with drift-line fence.

In addition to foot surveys, nine strategically placed drift-line fence traps were employed across the island in each habitat. Drift-line fence traps (Fig. 3) were particularly effective for detecting fossorial and cryptic species that are otherwise difficult to observe during visual surveys. Configured with a single funnel at one end and a double funnel at the other, each trap also incorporated a pitfall trap in the centre. These configurations were adapted to suit the varied terrain and the length of the funnel trap relative to the available area. With a 10-metre-long drift line, the traps provided an alternative means of detection for elusive, nocturnal or reclusive species.

Traps were strategically positioned across various habitats including the national park forest, human-disturbed forest and human settlements. They were deployed starting from 1 February 2022 and were checked daily over a period of 236 days. In addition to foot surveys, these traps provided an extended window for sampling snakes. Each snake encountered was promptly identified and measured at the field lab whenever possible before being released back into its natural habitat. The operational period of the traps coincided with the later stages of foot surveys, covering a duration from February 2022 to September 2023. Specific traps were left open for varying durations, ranging from 7 to 90 days.

Snake identification was facilitated through consultation of reputable sources such as Thailand’s Natural History Museum database (http://nhmsearch.nsm.or.th). To assess species diversity, we employed two ecological indices: the Shannon–Wiener [H' = −∑(pi × ln(pi))] and Simpson’s Diversity [D=1−∑(pi2]) (Krebs 1989). The Shannon–Wiener Diversity Index measures the diversity of a community by accounting for not only the species richness (the number of species), but also the evenness (how equally individual counts are distributed amongst the species). A higher Shannon–Wiener Index indicates greater diversity, characterised by both richness and evenness. We calculated the dataset with and without rescues (snakes removed from human–snake conflict situations) due to potential bias to some snake species, while also acknowledging the valuable insight into species most frequently encountered in human settlement areas. For the purpose of this paper, we used only survey, opportunistic and trap data. Occurrence data for all species recorded during surveys conducted between 2021–2023 on Ko Pha-ngan have been deposited in the Global Biodiversity Information Facility (GBIF) and are available at: doi: https://doi.org/10.15468/euvznr.

## Checklists

### Family Colubridae

#### Ahaetulla prasina

(Boie, 1827)

24C57204-19EB-509D-9D4B-F41A3A2624EA

##### Distribution

Known as the vine snake or whip snake, this species is primarily arboreal and detected easily throughout all three habitat types. A total of 52 individuals were detected with eight being from human settlements, 32 in human disturbed forest and 12 individuals were detected in NPF. Of the 52 individuals detected, 45 were surveyed and seven were opportunistic finds.

##### Notes

This arboreal species (Fig. 6) is diurnal; however, individuals were most frequently observed during night surveys while sleeping in trees, particularly in forest-edge habitats. This species feeds on a variety of prey items including frogs, lizards, small bird eggs, other snakes, birds and bats (Das et al. 2024). Notable characteristics include horizontal keyhole-shaped pupils allowing for acute binocular vision. Black and white patterned skin between scales which is visible when the snake is in defensive display. Neonates observed were a pale brown or tan colour and adults were green, despite other colour morphs known in this species (Lillywhite and Martins 2014, Lalremsanga 2022). Being ovoviviparous, *A. prasina* gives birth to live young (Lalremsanga 2022). One gravid female was observed on 2 August 2023.

#### Boiga cyanea

(Duméril, Bibron et Duméril, 1854)

9DFD7F13-81C7-5B6A-B066-7FA9589C6AFD

##### Distribution

A total of 18 individuals were detected across the three habitats. One individual was detected in human settlement (surveyed), seven individuals were detected in human disturbed forest (surveyed) and ten individuals in NPF (one trapped and nine surveyed).

##### Notes

Known as the green cat-eyed snake, this species (Fig. 7) is nocturnal and primarily arboreal preying upon birds, small bird eggs, lizards and frogs (Greene 1989, D’souza et al. 2021). This snake is oviparous (two gravid females observed 23.01.22 and 29.05.23) and considered mildly venomous though not medically significant to humans. Notable features of this snake included a black mouth when open, head is distinct from the neck, head length is greater than head width, vertically elliptical pupils, green dorsal (along the back) scales with yellow ventral scales (along the belly) (D’souza et al. 2021).

#### Chrysopelea ornata

(Shaw, 1802)

B582BEFE-C581-573E-8934-24D52F64F870

##### Distribution

A total of eight individuals were detected across all habitat types and all were opportunistic detections. Of the eight, three were detected in human settlements, two were detected in human disturbed forest and three in NPF areas.

##### Notes

Commonly referred to as the ornate flying snake, this species (Fig. 8) is diurnal, oviparous (gravid female observed 8 February 2022) and arboreal with a unique ability to glide from tree to tree by flattening its body (Socha 2011). The diet consists of a variety of lizards, frogs, birds, bats and small rodents (Diesmos et al. 2004, Correa et al. 2023, Mithu et al. 2024). Dorsal scales form a vivid black pattern over a bright yellow to green background, with the colour and pattern varying amongst individuals (Chan-ard et al. 2015).

#### Coelognathus radiatus

(Boie, 1827)

8F795BD6-9FD3-5F38-BA92-51E1F45ADE1B

##### Distribution

Five individuals were observed spanning all three habitat types. Three individuals were detected in human disturbed forest (three opportunistically) and two in NPF (two via traps).

##### Notes

Commonly known as the radiated rat snake, this species (Fig. 9) is diurnal and oviparous (two observed gravid females on 7 July 2021 and 11 December 2021). Notable characteristics of this snake include a transverse black occipital line radiating from the eye and plain ventral scales often cream in colour (Gumprecht 2003). This species feeds on rats, mice, a variety of frogs, lizards and some birds. As defensive behaviour, *C. radiatus* exhibits thanatosis or death feigning when stressed (Nadolski et al. 2020).

#### Dendrelaphis caudolineatus

(Gray, 1834)

2D4128BE-8DE8-5787-9C91-64BBA0C97A50

##### Distribution

Only one individual was detected on a night survey in NPF (observed gravid 14 October 2022). After the study period concluded, one more individual was found dead on the road and is not included in the data represented here; however, we found it important to note as it was within a few kilometres of the first one.

##### Notes

Commonly known as the striped bronzeback (Fig. 10), this is arboreal and diurnal with a diet consisting of lizards and frogs (Van Rooijen and Vogel 2012). This snake includes the quintessential large eyes for the genus *Dendrelaphis* with notable characteristics including bronze-brown or reddish-brown dorsal colouration with lateral strips along the body with a distinct cream, yellow or orange stripe along the flank.

#### Lycodon capucinus

Boie, 1827

B21296DC-7C34-5F43-B262-AFD79AC38CA0

##### Distribution

A total of 18 individuals were detected with four individuals recorded in human settlements (survey), eight individuals were detected in human-disturbed forest areas (survey) and six individuals recorded in NPF areas (via traps).

##### Notes

The common wolf snake (Fig. 11) is a nocturnal species, well adapted to human settlement areas and often found in and around homes including the author’s house on two different occasions. Diet consists of geckos, skinks and sometimes small frogs and mice. Notable characteristics of this snake include a flattened almost shovel-like head, brown dorsal colouration with cream or yellow speckles throughout the body. Often with a collar at the base of the head. This snake is rear-fanged and semi-arboreal (Fritts 1993, Jackson and Fritts 2004). This oviparous snake was observed gravid on four separate occasions (19.09.21, 08.03.22, 09.04.22, and 28.04.22). This species is distributed throughout the entire island in all habitat types.

#### Lycodon davisonii

(Blanford, 1878)

0C5CCA0C-24B5-5BB0-ACAF-C56335084135

##### Distribution

A total of five individuals were detected. Three in human disturbed forest (survey) and two in NPF (survey).

##### Notes

The bidle snake (Fig. 12 ) is nocturnal and considered semi-arboreal. It is a top predator of small bird eggs, also preying upon geckos and other small vertebrates (Khamcha and Gale 2020). Distinguishing features of this slender snake include dark saddles with white bars interspersed. This snake is oviparous and one individual was observed gravid on 26.09.22.

#### Oligodon phangan

(Pauwels, Thongyai, Chantong et Sumontha, 2021)

CD859D0B-7EA4-57A6-8696-B2CEB5874638

##### Distribution

Seven individuals were detected. Two in human disturbed forest (two in survey) and five in NPF (two in trap and three in survey).

##### Notes

Endemic to Ko Pha-ngan, the Phangan kukri is nocturnal and semi-fossorial, this species being observed eating reptile eggs (Pauwels et al. 2021). Distinguishing features of this snake include faint paravertebral and lateral stripes slightly more pronounced in neonates due to the darker colouration, fading as the snake reaches adulthood (Fig. 13). Dorsal (back) is brown to coral in colouration, while ventral scales (belly) are a more pronounced pink to orange in colour. This species engaged in male combat (Shine 1978, Shine et al. 1981, Huang et al. 2011, Senter et al. 2014) which was opportunistically observed on 31.12.2022 behind a private residence.

#### Ptyas korros

(Schlegel, 1837)

703FC48F-1D2F-5032-A645-FD0C0515E9FB

##### Distribution

Five individuals were detected opportunistically in human disturbed forest areas.

##### Notes

The Indo-Chinese rat snake (Fig. 14) is diurnal with a diet consisting of small rodents, frogs and lizards. Primarily terrestrial, *P. korros* can also be seen in low vegetation. Distinguishing features of this snake are large eyes and smoky grey/brown/olive dorsal colouration with plain cream to yellow ventral scales (Platt et al. 2017).

#### Rhabdophis chrysargos

(Schlegel, 1837)

164D56DD-B67C-58C4-B099-C0963C2404E7

##### Distribution

A total of two individuals were detected in one transect in NPF (two surveyed).

##### Notes

Also known as the speckle-bellied keelback, this species (Fig. 15) is diurnal oviparous and considered semi-aquatic, commonly found near forest streams, rice paddies and wetland margins within lowland and disturbed evergreen forests. It primarily preys on frogs and tadpoles, occasionally consuming small fish and lizards (Chan-ard et al. 2015, Harman and Master 2021). Distinguishing characteristics include strongly-keeled dorsal scales, a light (often white or yellowish) chevron marking immediately posterior to the head and dorsal colouration of olive to brown with dark crossbands that often form broken chevrons along the body. The ventral scales (belly) are speckled or mottled and the supralabial scales are lined so that it looks like black broken bars along the labials (around the mouth).

#### Xenochrophis triangulagaris

(Heinrich Boie, 1827)

5E87258C-D603-5358-A178-52A0680ED694

##### Distribution

This species was detected only once. One individual in one transect in NPF (one survey).

##### Notes

The triangle keelback (Fig. 16) is considered diurnal (often more active around dusk or dawn) and semi-aquatic primarily preying upon a variety of frogs and has been known to consume fish and lizards on occasion. Distinguishing characteristics of this species include keeled scales, olive in colouration with black evenly-spaced markings often triangular in shape. Flanks may be red-yellow or red-orange dulling in colour moving towards the tail. Ventral scales are light with some dorsal colouration bleeding down into the ventral scales causing intermittent complete and incomplete markings on cream-coloured scales. This snake is oviparous and observed gravid on 29 July 2023.

### Family Cylindrophiidae

#### Cylindrophis jodiae

Amarasinghe, Ineich, Campbell et Hallermann, 2015

2960AB93-B5CF-5455-AB3C-57A43739369E

##### Distribution

We detected eight individuals across the three habitat types: six in three different human settlement areas, three in two of the thirteen human disturbed forest transects and one from one of eleven NPF transects. Of the eight individuals detected, three were by survey, four opportunistically and one was trapped.

##### Notes

Also known as the pipe snake, this species (Fig. 5) is cylindrical in shape and has a distinguished defensive behaviour in which its red tail is raised above the body in an apparent strike position. This snake is non-venomous and fossorial (primarily living underground) with a diet consisting of worms, eels, grubs and small snakes. Notable characteristics of this snake include brightly coloured (red or orange) interrupted dorsal bands dulling and darkening by adulthood, alternating black and white ventral scales (belly scales) creating a checkered pattern. This snake is ovoviviparous (Amarashinghe et al. 2015, Bernstein et al. 2020, Das et al. 2024).

### Family Elapidae

#### Calliophis maculiceps

(Günther, 1858)

BD51FB06-D5E4-5136-A1E9-3F244EC535C0

##### Distribution

Two individuals were detected in human settlements via night surveys and one in NPF opportunistically during the day.

##### Notes

Known as the small spotted coral snake, this snake is nocturnal and is often found under leaf litter in sandy areas often near streams. Distinguishing features of this species are slender with distinct spots or blotches running along its side (dorso-laterally). Ventral scales are often pink or coral in colour with the subcaudal scales being pale with black bands often displayed when threatened (Fig. 19; Yamasaki et al. (2001)). The snakes examined on Ko Pha-ngan did not display the small “spots” traditionally present on this species. A total of three individuals were detected in two of the three habitats.

#### Naja kaouthia

Lesson, 1831

A523AFB1-0637-50C3-8C09-1EAD97D964BF

##### Distribution

A total of three monocled cobras were detected throughout all three habitats. One opportunistic detection in each habitat.

##### Notes

Known as the monocled cobra (Fig. 20) and is considered diurnal; however, it is often detected moving in the late-night hours and on three different occasions, this activity was captured on camera traps. Diet consists of a variety of amphibians, lizards and rodents. Notable features of this snake include the iconic “monocled”-shaped hood marking presented when exhibiting defensive behaviour.

#### Ophiophagus bungarus

Schlegel, 1837

39BC6399-C09E-595F-B124-B290B77ACF2F

##### Distribution

A total of three individuals were detected throughout the island. One in human disturbed forest (one opportunistic) and two in NPF (two opportunistic).

##### Notes

The king cobra's diet primarily consists of snakes; however, occasionally it is known to consume monitor lizards (Bhaisare et al. 2010, Kurniawan et al. 2018). Apart from its iconic hood (Fig. 21) and large size, defining features of the king cobra include the presence of distinct occipital scales which are unique and can easily differentiate them from true cobras. Colouration can vary from a lighter tan or olive brown to a darker brown or black, often with lighter crossbands, chevron in shape, on the neck towards the head smoothing out moving down the body and eventually fading. The tail is generally an even darker olive brown with scales seemingly outlined in black creating a unique pattern distinct for the king cobra; however, as an adult *Ptyas korros* and *Ptyas mucosa* (not on the island) have similar tail scale colouration and patterning. This species went through a taxonomic revision and recent work by Das et al. (2024) resurrected *O. bungarus* (Schlegel 1837) for Sundaic populations, while restricting *O. hannah* (Cantor 1836) to mainland Indochina and India.

### Family Homalopsidae

#### Homalopsis buccata

(Linnaeus, 1758)

D73363EC-EDAC-5E97-856F-A262652A4705

##### Distribution

A total of seven individuals were detected in two human settlement areas.

##### Notes

Known as the puff-faced water snake, this species (Fig. 17) is nocturnal, semi-aquatic and considered mildly venomous using its venom to subdue prey which primarily consists of a variety of fish and frogs and even crustaceans (Fabre et al. 2016). This snake is partial to fresh water; however, murky slow-moving streams are favoured along with other freshwater bodies such as canals (klongs) and small ponds. It is also known to be a prey item for *Cylindrophis jodiae* (Voris and Murphy 2002). Distinguishing features of this snake include heavily keeled and striated scales, moon or crescent-shaped closable or valved nostrils and a dark brownish-black banded pattern which fades as it ages. This snake is ovoviviparous (Berry and Lim 1967) as they do not lay eggs; however, they retain their young which hatch inside the mother and then she births the live young (Berry and Lim 1967). One individual was observed gravid (26 June 2022) and she gave birth to 36 young with 22 being viable.

### Family Pareidae

#### Pareas margaritophorus

(Jan, 1866)

44C7AE0C-4AC3-5BC4-AA89-17F363BF0E2D

##### Distribution

All three documented individuals were found in NPF, in two of eleven NPF transects. Two were captured by trap and one was observed opportunistically on a rock adjacent to a trap.

##### Notes

**Figure 4.**
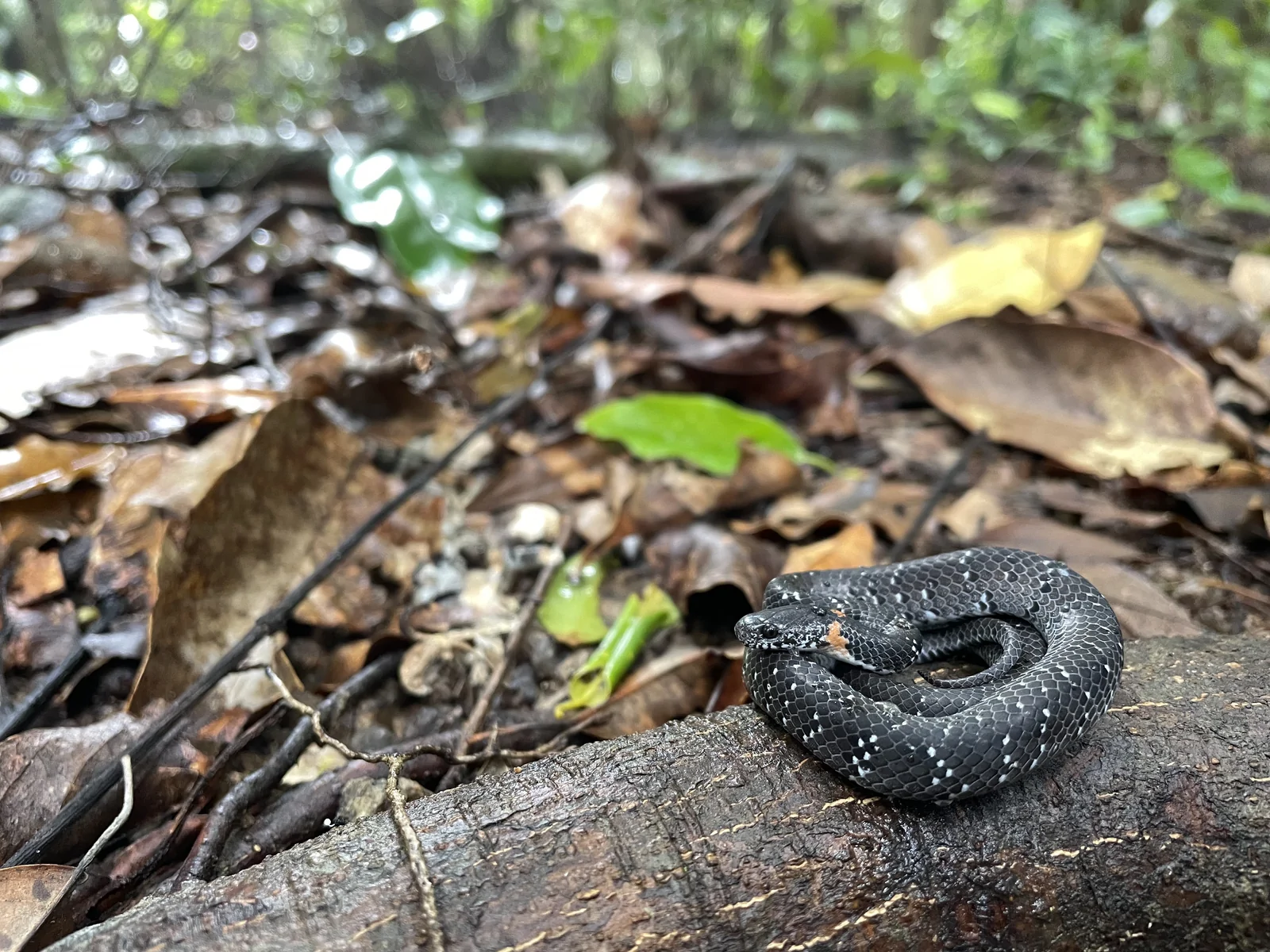
*Pareas margaritophorus* at national park forest trap site.

**Figure 5.**
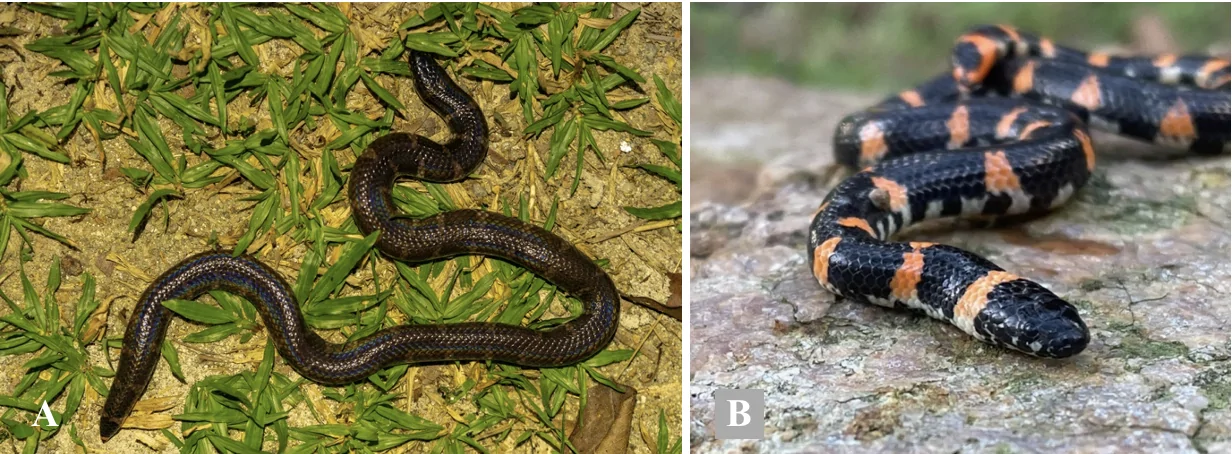
*Cylindrophis jodiae*. **A** From above (dorsal view); **B** juvenile colouration.

**Figure 6.**
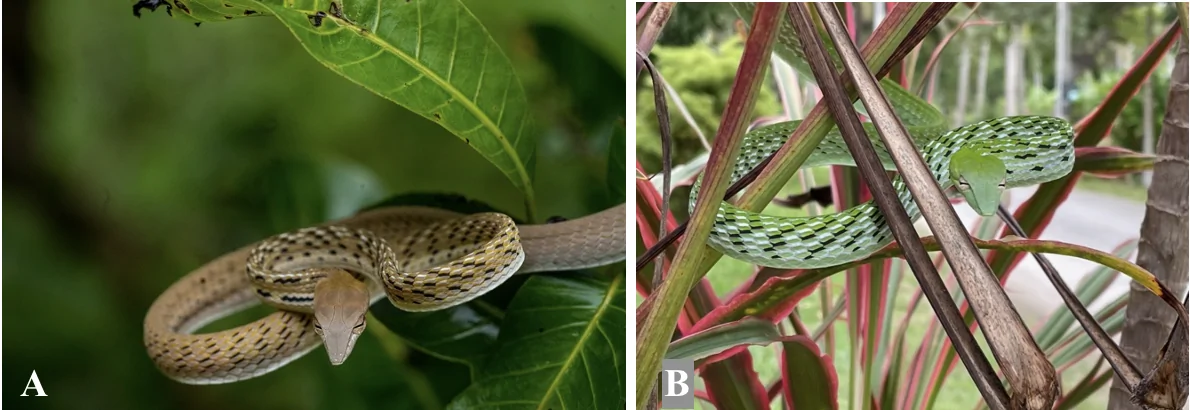
*Ahaetulla prasine*. **A** Juvenile brown colouration; **B** adult green colouration.

**Figure 7.**
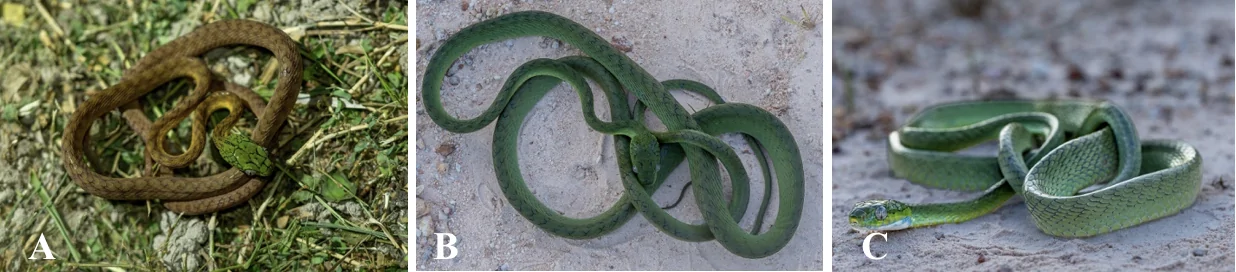
*Boiga cyanea*. **A** Adult front head view; **B** adult side view; **C** juvenile.

**Figure 8.**
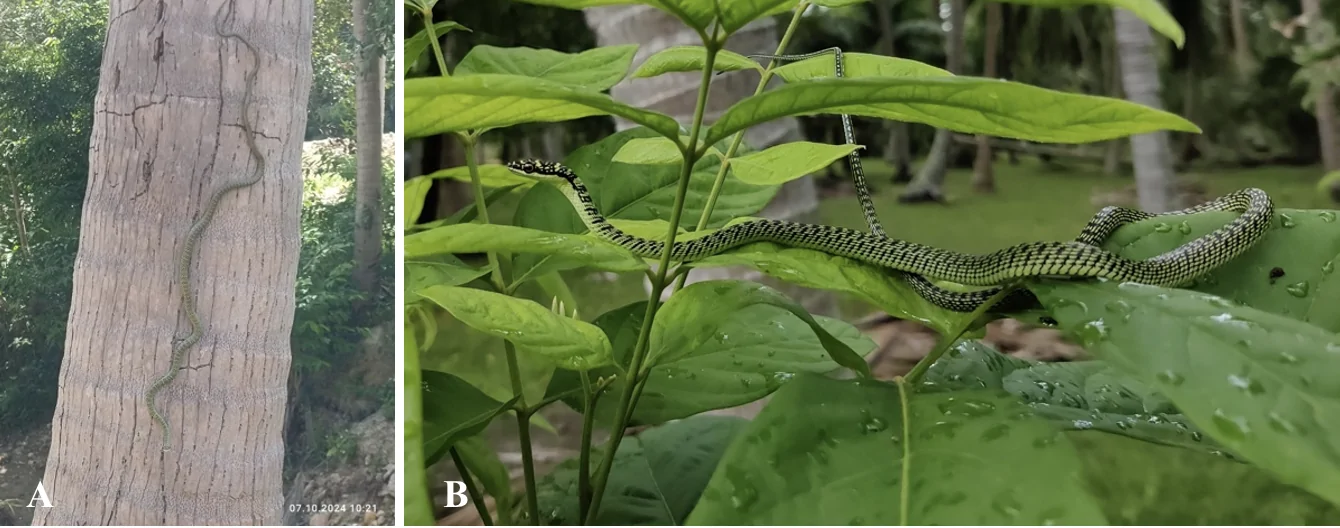
*Chrysopelea ornata*. **A** Moving down a coconut tree and **B** moving through low vegetation.

**Figure 9.**
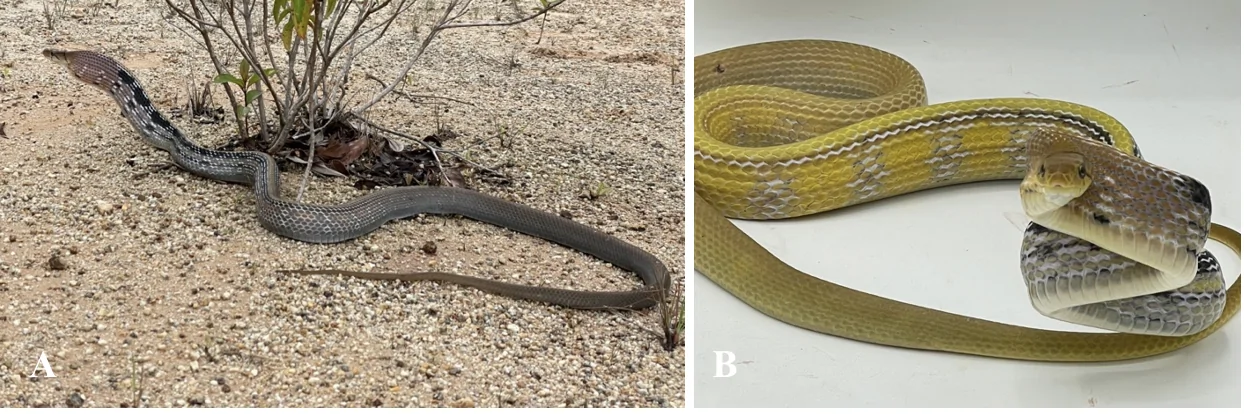
Two different *Coelognathus radiatus* colour variations. **A** Brown/tan typical colouration; **B** the rarer yellow colouration (gravid female).

**Figure 10.**
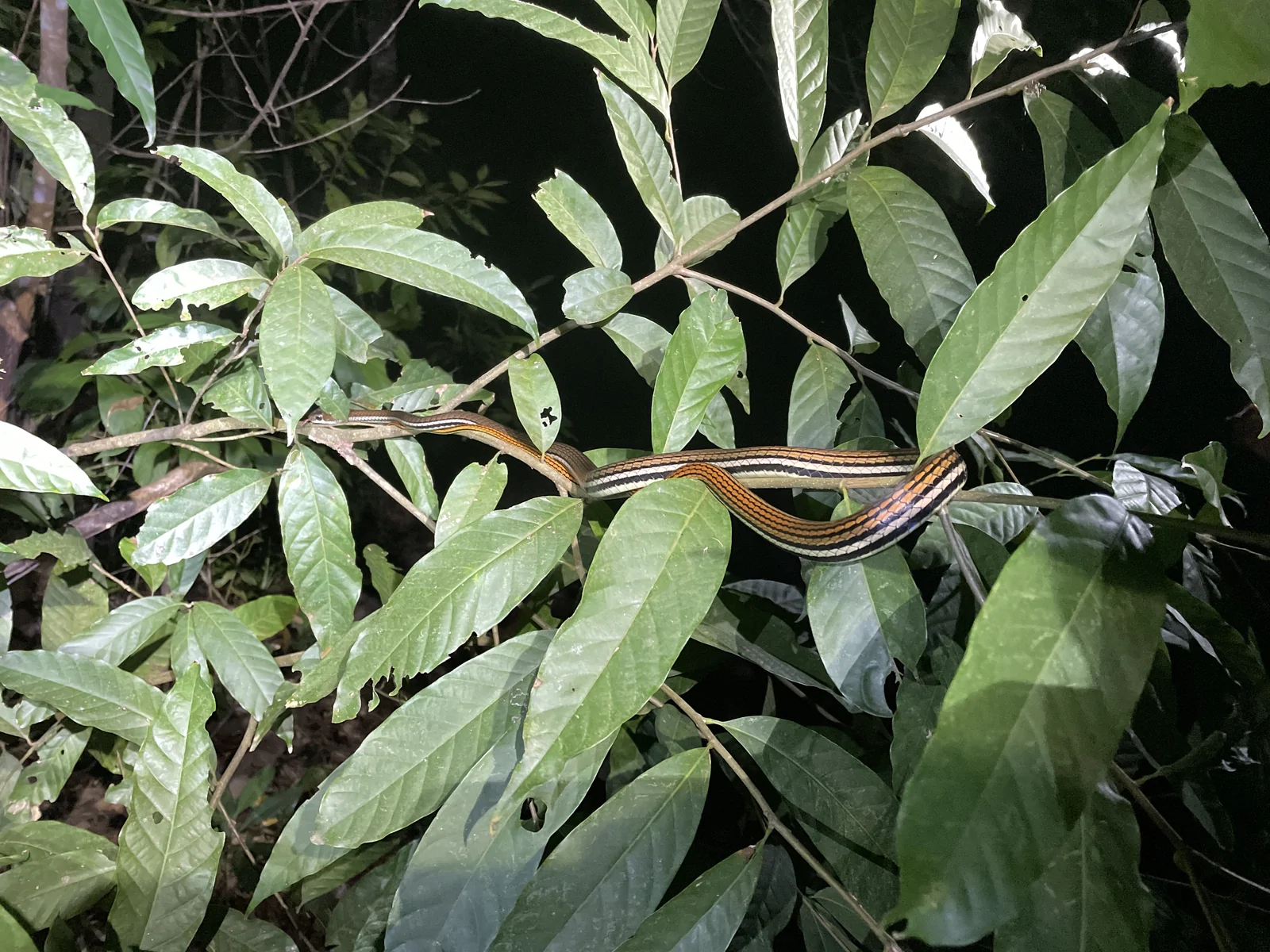
*Dendrelaphis caudolineatus* in situ.

**Figure 11.**
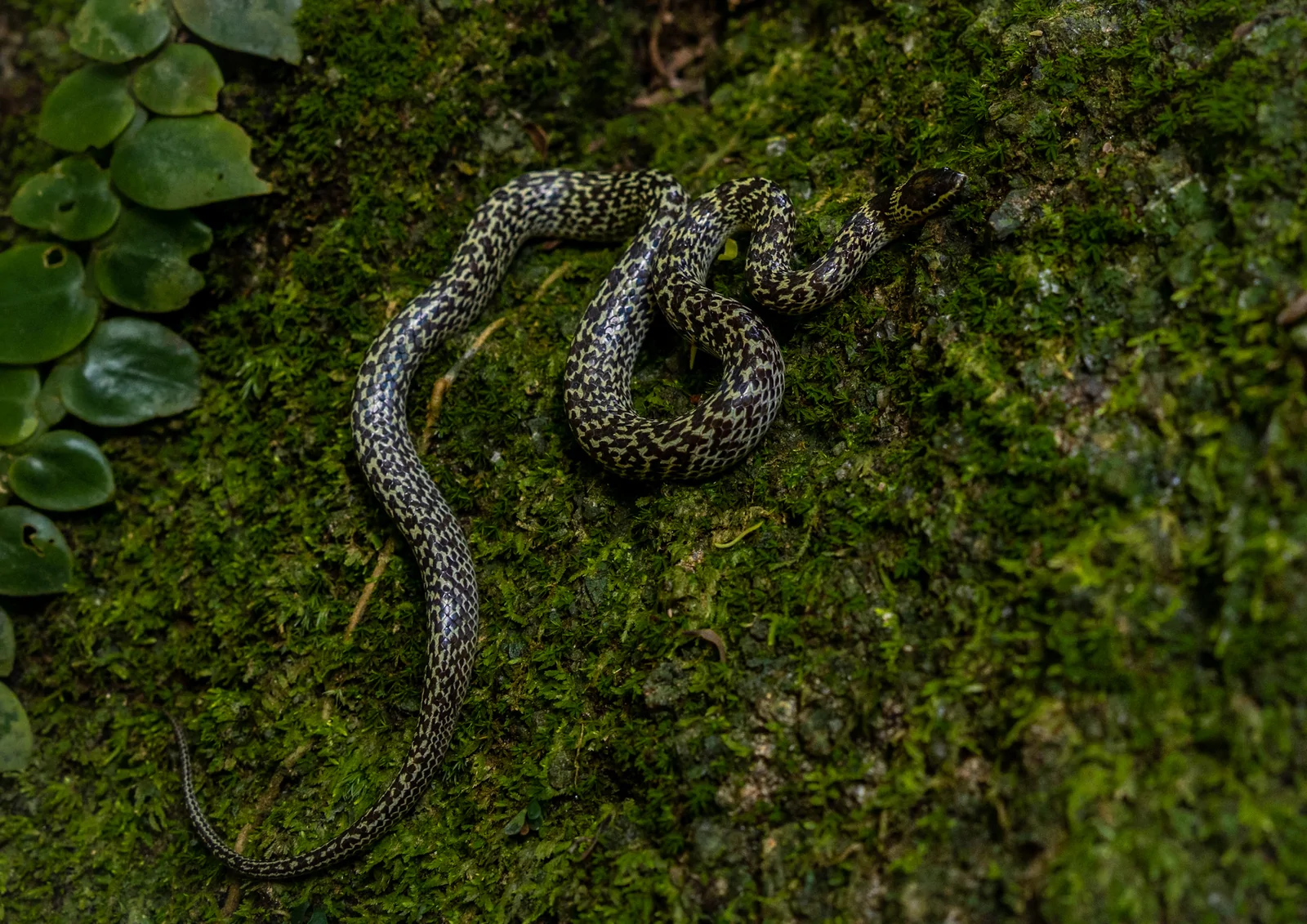
*Lycodon capucinus* on moss covered rock at NPF trap site.

**Figure 12.**
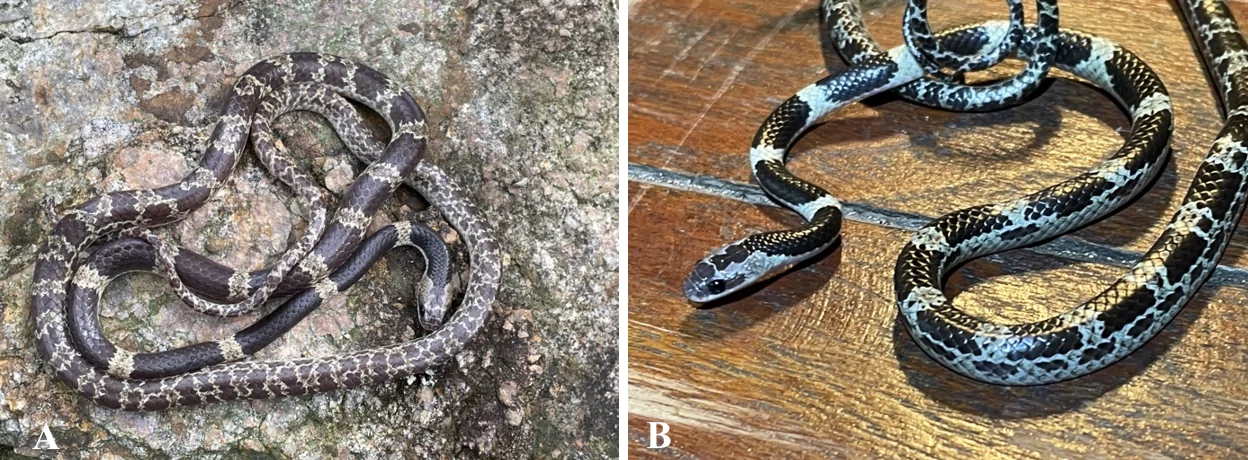
*Lycodon davisonii*. **A** brown and cream colouration; **B** black and white colouration.

**Figure 13.**
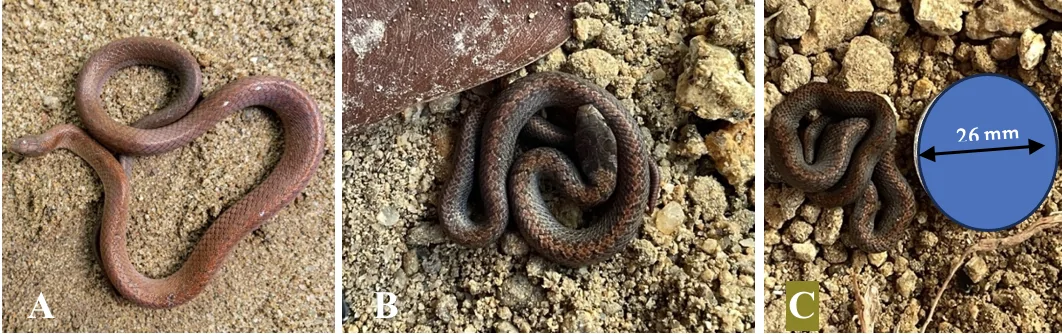
*Oligodon phangan*. **A** adult; **B** neonate; **C** neonate with size reference.

**Figure 14.**
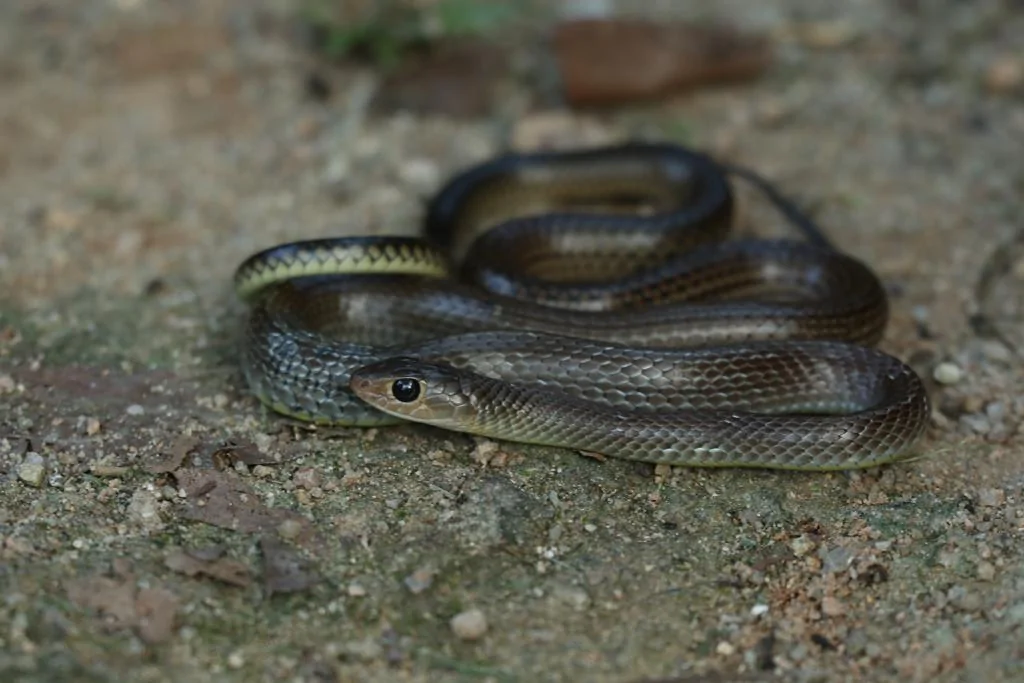
*Ptyas korros*.

**Figure 15.**
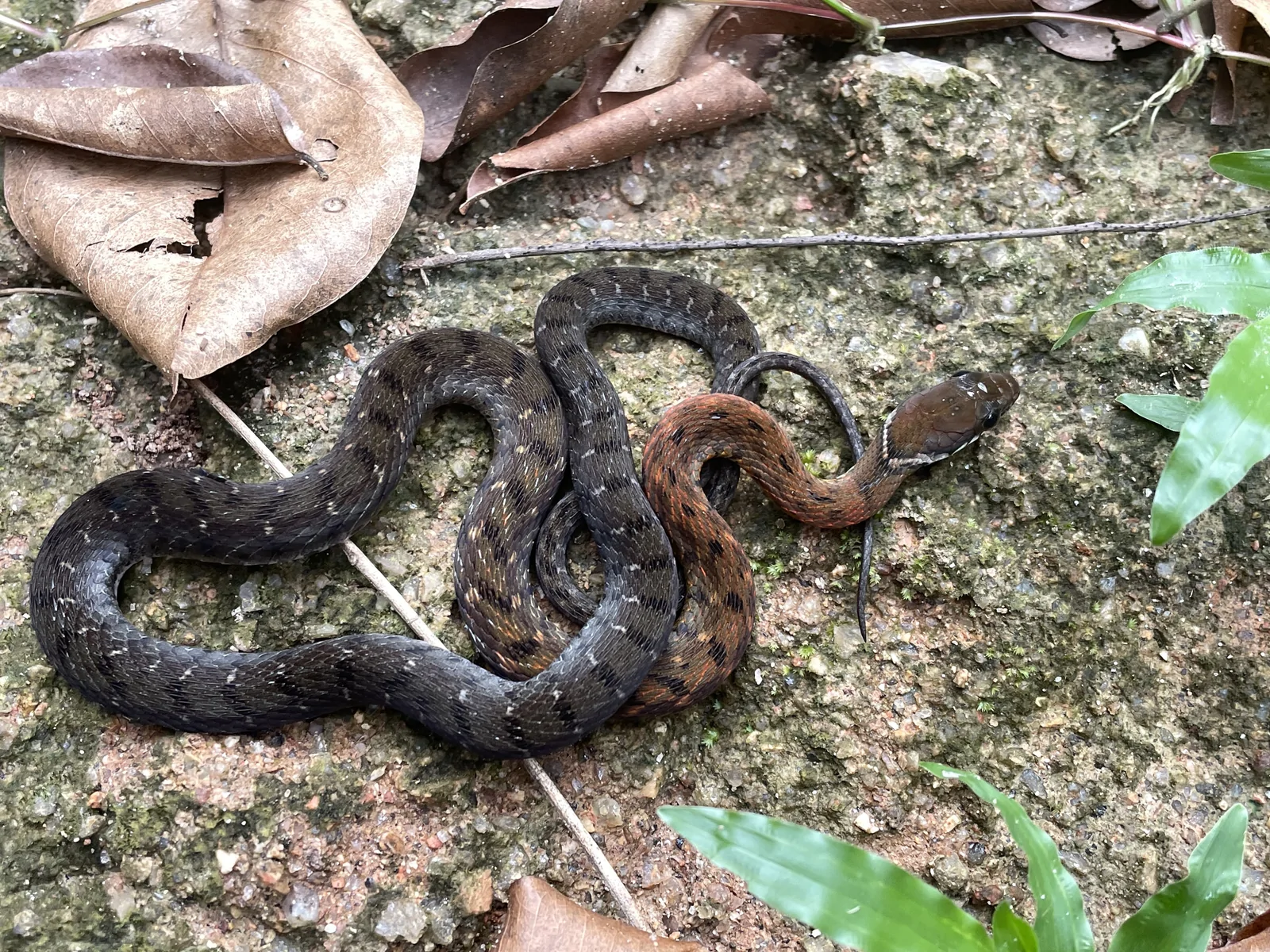
*Rhabdophis chrysargos*.

**Figure 16.**
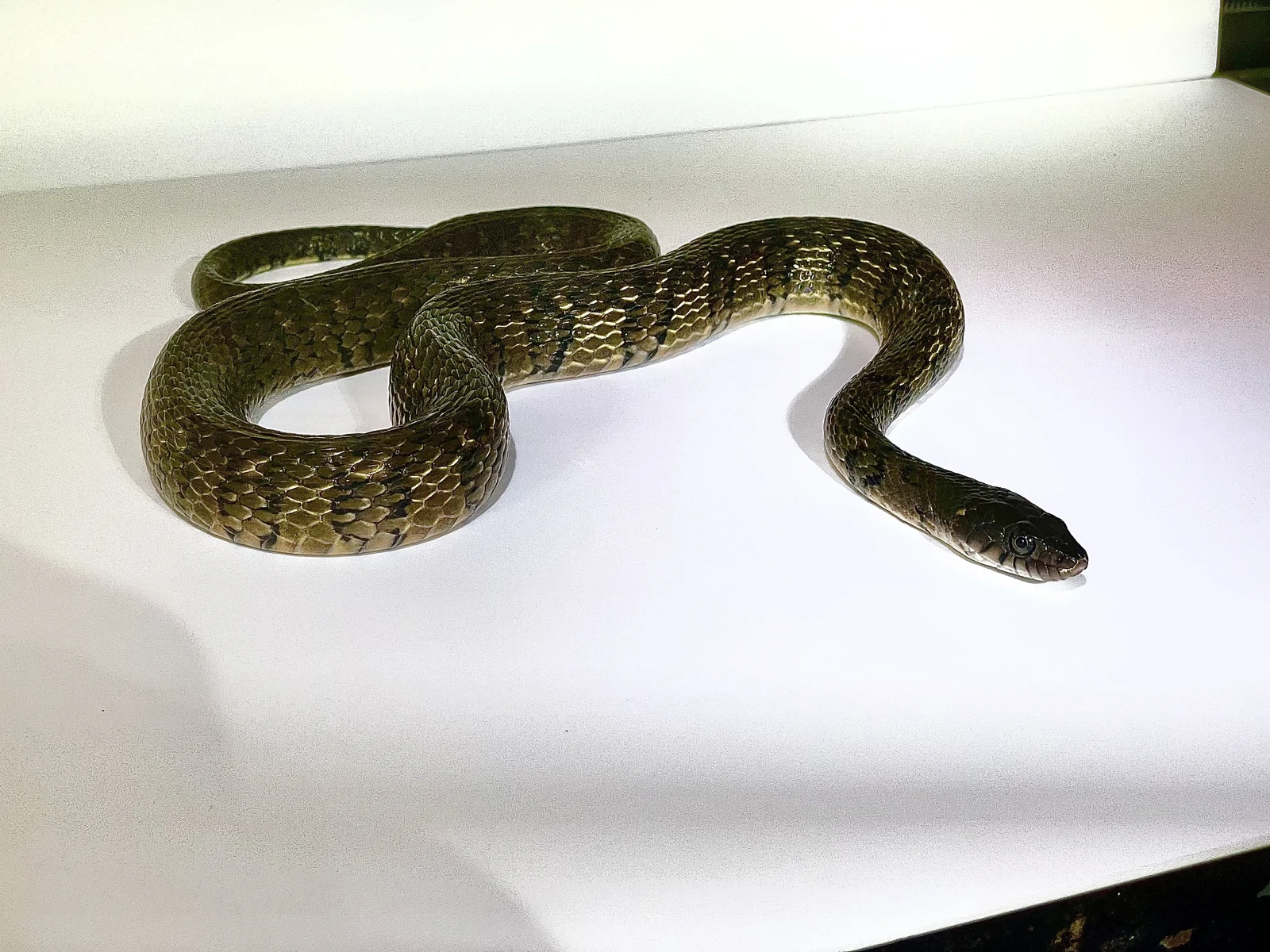
*Xenochrophis trianguligerus*.

**Figure 17.**
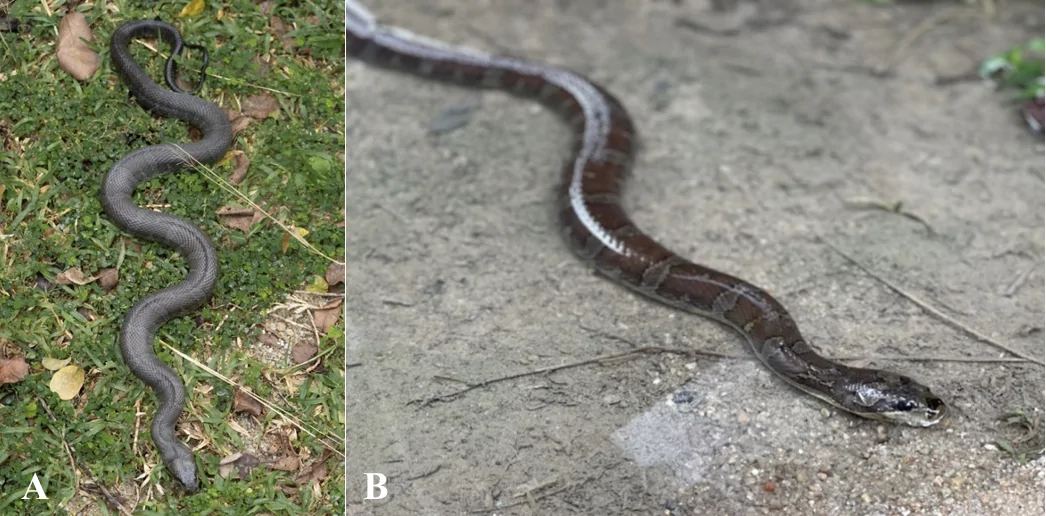
*Homalopsis buccata*. **A** dorsal view; **B** side view with colouration noticeable.

Commonly known as the white spotted slug snake, this nocturnal pareid (Fig. 4) is a specialist, utilising mandibular transport when consuming slugs and snails (Götz 2021). Primarily fossorial occupying the forest floor, this snake is phenotypically similar to the mountain slug snake (*Pareas macularius*); however, *Pareas margaritophorus* can be differentiated by smooth scales and a cream to pinkish nuchal collar, whereas *P. macularius* possesses keeled dorsal scals and a distinctive whitish or brown W-shaped (butterfly-shaped) nuchal collar (Hauser 2017). This snake is oviparous (egg laying) and one female was observed gravid with four ova detected (8 March 2022).

### Family Pythonidae

#### Malayopython reticulatus

(Schneider, 1801)

7D5AE792-0AEC-5CB8-9B07-DCEBB9030D20

##### Distribution

Nine individuals were detected across all habitat types. Three in human settlement areas (1 survey, 2 opportunistic), four in human disturbed forest areas (two survey and two opportunistic) and two in NPF (two survey).

##### Notes

**Figure 18.**
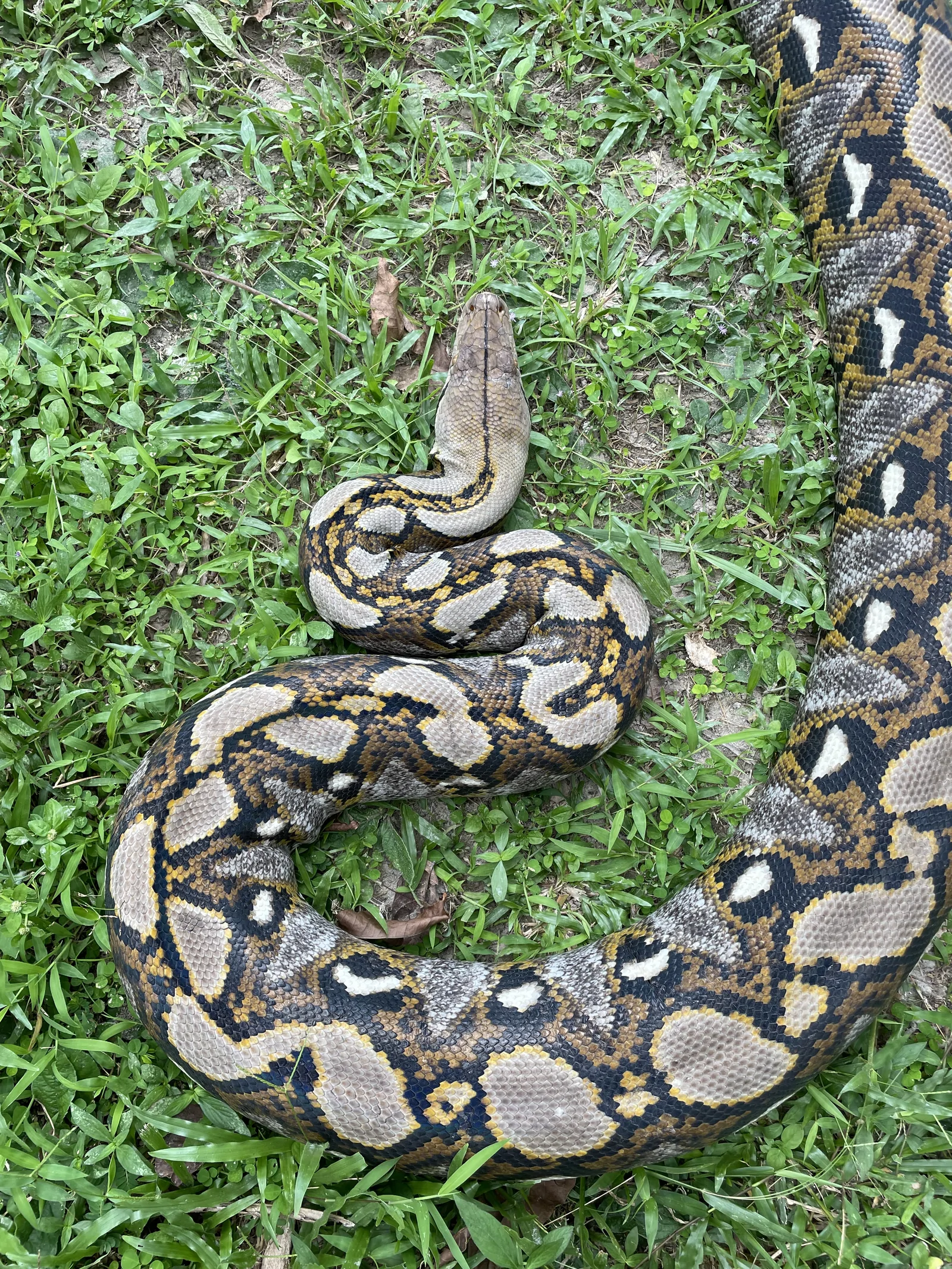
*Malayopython reticulatus*.

**Figure 19.**
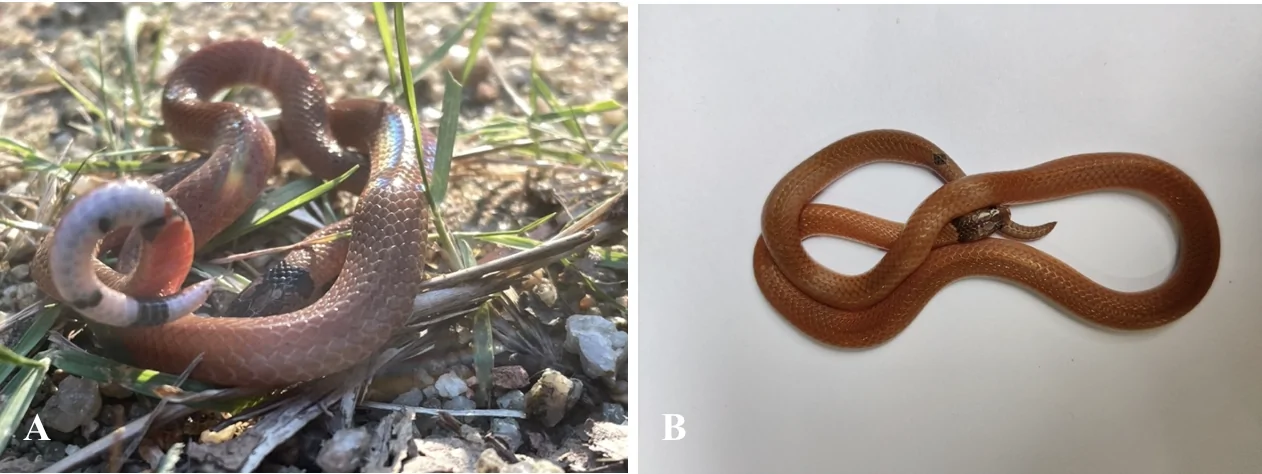
*Calliophis maculiceps*. **A** defensive posture; **B** dorsal view.

**Figure 20.**
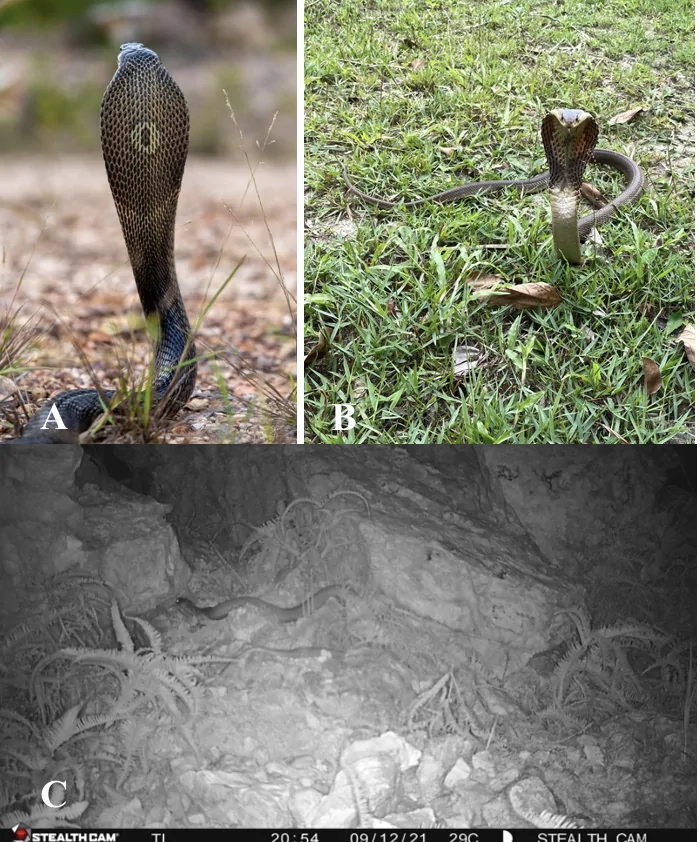
*Naja kaouthia*. **A** back hood marking; **B** defensive posture; **C** moving out of a burrow.

**Figure 21.**
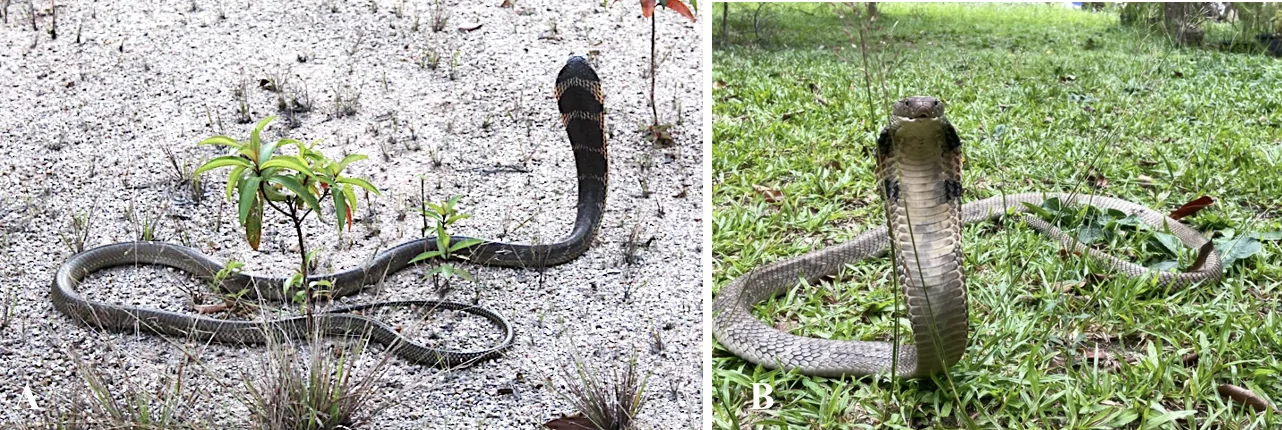
*Ophiophagus bungarus*. **A** chevron pattern on the dorsal hood; **B** front defensive posture.

The reticulated python (Fig. 18) is nocturnal. It is an opportunistic constricting ambush predator; however, it can be quite active and is often in conflict with villagers due to easy prey opportunities by way of kept chickens. Diet consists of a variety of prey items including mammals ranging in size as the snake grows. Primarily rats, bats, squirrels, chickens, feral cats, civets, macaques, wild pigs and deer all found on Ko Pha-ngan Island (Cook-Price et al. 2025). Options are limited on this island as the mammal populations are considerably different from the mainland populations. Differentiating features of this species include both supralabial and infralabial thermal receptive pits and a dorsal lateral reticulated or net-like pattern (Burger 2022).

### Family Typhlopidae

#### Indotyphlops braminus

(Daudin, 1803)

34453A53-55D1-59C4-9BE0-ABCE6FBC435A

##### Distribution

Only one individual was detected in human settlement areas (opportunistic).

##### Notes

**Figure 22.**
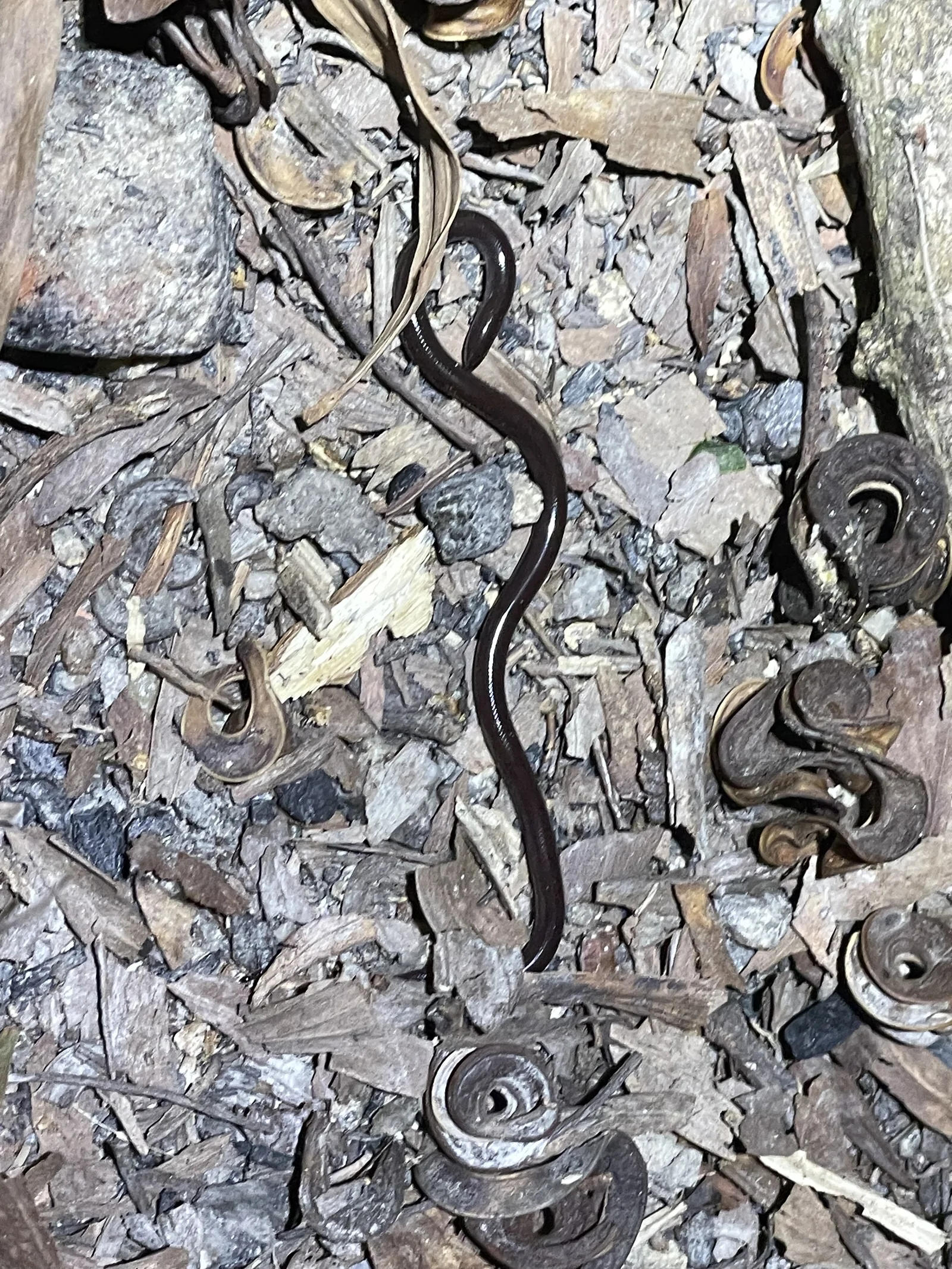
*Indotyphlops braminus*.

Commonly known as the Brahminy blind snake, its diet primarily consists of larvae, ants and termites. Fossorial in nature, this snake can be found from garden soil in human settlements (Fig. 22) to under leaf litter throughout the forest floor. Found throughout the world, *I. braminus* is also known as the flowerpot snake as it has been human introduced globally via flora shipped from nation to nation. Defining features of this snake include distinct scale junctions known to this species such as the downward curving infranasal suture (the lines formed where the scales join around the nostril) extending from the nostril until touching the preocular scale. This snake is parthenogenetic as females reproduce independently from males.

#### Argyrophis muelleri

(Schlegel, 1839)

1E5DD13D-713D-5AE7-A166-1B102043F7B5

##### Distribution

Only one individual was observed opportunistically next to the funnel trap in one NPF trap area.

##### Notes

**Figure 23.**
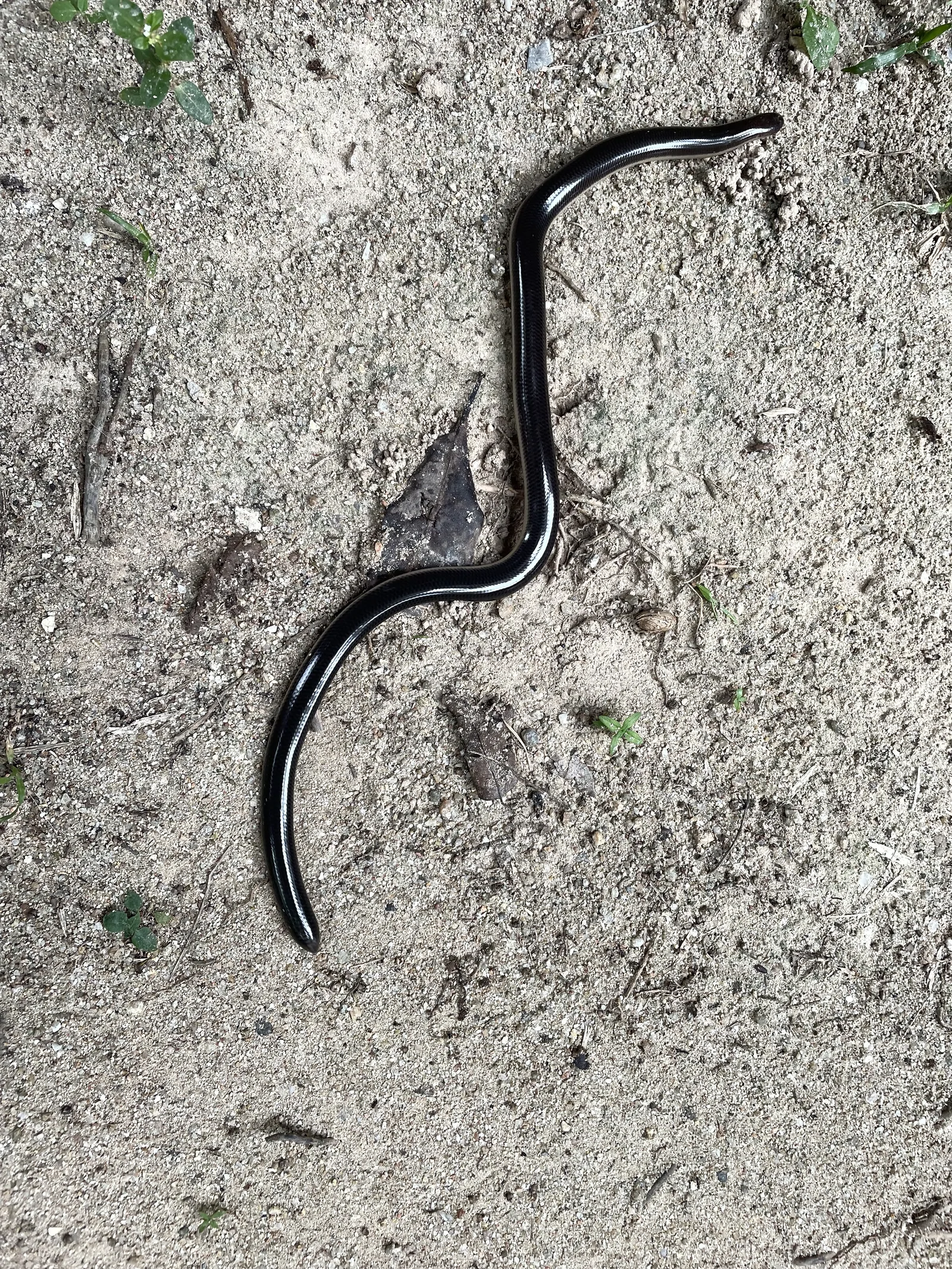
*Argyrophis muelleri*.

This species (Fig. 23) is one of the largest blind snakes in Thailand, commonly known as the white-bellied blind snake. Fossorial and preys upon pupae and larvae of both ants and termites as well as a variety of worms, soft insects and even smaller snakes. Notable characteristics of this snake include the abrupt and distinct change between the dark brown or purplish-black dorsum to the cream or white venter (Wallach 2001, García-Antón et al. 2022).

### Family Xenopeltidae

#### Xenopeltis unicolor

(Reinwardt, 1827)

8B18D683-A90A-51A6-8F25-96842B88D76E

##### Distribution

A total of eleven individuals were detected in two of the eleven NPF transects, two of the thirteen NPF transects and one of the human disturbed forest transects. All were detected by survey.

##### Notes

**Figure 24.**
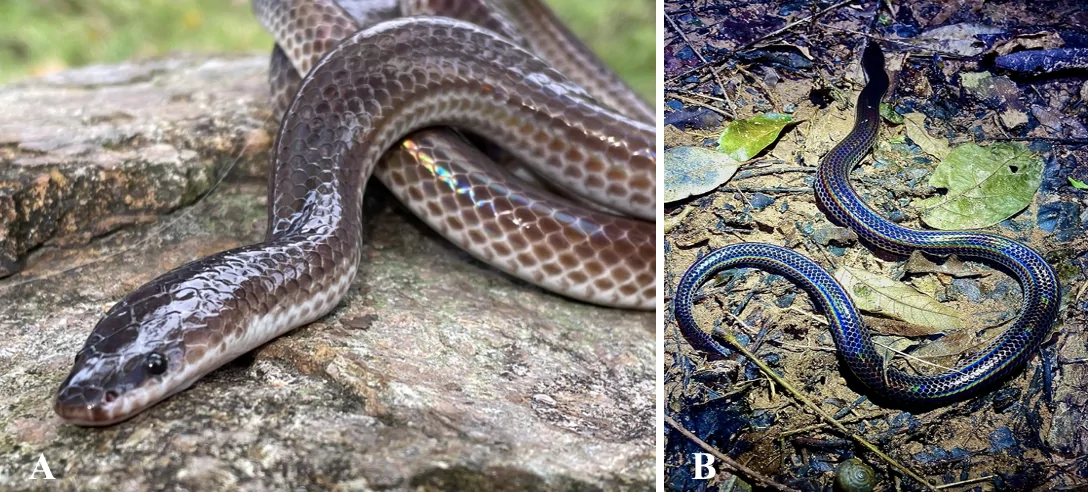
*Xenopeltis unicolor*. **A** lighter colouration; **B** darker colouration, dorsal view.

The sunbeam snake (Fig. 24) is nocturnal and fossorial, preying upon frogs, lizards, other snakes and small mammals. Notable features of this snake include highly iridescent dark brown dorsal scales (back) fading to white. Ventral scales (belly) are completely white.

## Analysis

A total of 21 snake species were documented throughout the duration of this study on Ko Pha-ngan (Table 1). Representing 20 genera across eight families: Pareidae (1 species), Cylindrophiidae (1 species), Homalopsidae (1 species), Pythonidae (1 species), Elapidae (3 species), Typhlopidae (2 species), Xenopeltidae (1 species) and Colubridae (11 species). We were able to add an additional 17 species to the existing records maintained by the Department of National Parks, Wildlife and Plant Conservation (2018), significantly enriching the official record. Newly-recorded species include *P. margaritophorus*, *C. jodiae, A. prasina*, *B. cyanea*, *C. ornata*, *D. caudolineatus*, *L. capucinus*, *L. davisonii*, *O. phangan*, *P. korros*, *R. chrysargos*, *X. trianguligerus*, *H. buccata*, *M. reticulatus*, *C. maculiceps*, *I. braminus*, *A. muelleri* and *X. unicolor*.

Amongst the recorded species, two stand out as conservation concerns: *P. korros* which is listed as Near Threatened and *O. bungarus* which is listed as vulnerable with numbers decreasing according to the International Union for Conservation of Nature and Natural Resources Red List (IUCN 2024). The island also contains one endemic species of snake, *O. phangan*.

Our analysis revealed that, of the 171 individual snakes detected throughout the island, the most diverse habitat was NPF habitat (Table 2). Twenty species were detected in NPF habitat followed closely with 11 in HS areas and 13 in HDF, respectively.

National Park Forest exhibited the highest Shannon–Wiener diversity (2.61), followed by human settlement (2.10) and human-disturbed forest (1.96) (Table 2). Simpson's diversity (1−λ) showed the same pattern, with National Park Forest having the highest diversity (0.898), followed by human settlement (0.859) and human-disturbed forest (0.779). Visual surveys accounted for nearly 70% of all detections, opportunistic encounters contributed over 23% and drift-line traps yielded approximately 8% of records (Table 1). The most frequently detected species were *A. prasina*, *B. cyanea*, *X. unicolor* and *L. capucinus*, with 45, 17, 12 and 11 encounter records, respectively. Seven species occurred in all three habitat types, whereas six species were restricted to a single habitat; five of the six (*P. margaritophorus*, *D. caudolineatus*, *R. chrysargos*, *A. muelleri* and *X. trianguligerus*) were recorded exclusively from National Park Forest.

Visual surveys were the most effective detection method, accounting for nearly 70% of all records (Table 1). Opportunistic encounters added over 23%, demonstrating their importance in detecting species outside regular survey periods (especially arboreal and nocturnal taxa). Trapping yielded only 8%, indicating limited effectiveness.

Detectability likely influenced the observed patterns as arboreal species and cryptic fossorial (burrowing) species are inherently challenging to spot, potentially skewing results. For instance, *N. kaouthia* may well inhabit the national park forest habitat; however, it is more readily encountered and reported in human settlement due to their large size and potential danger. In contrast, some habitats, such as edge habitats, provide improved visibility, favouring easier detectability. In addition, some fossorial species, such as *C. jodiae*, might not have been detected in human settlement areas save for gardening projects that involved digging or moving soil. The heightened snake diversity in national park forest regions is potentially attributed to the habitat being the least disturbed of the three.

We found the most common snake species from surveys to be *Ahaetulla prasina, Boiga cyanea*, *Xenopeltis unicolor* and *Lycodon capucinus*, as 45, 17, 12 and 11 individual sighting occasions, respectively (Table 1). As many habitats face destruction, certain species demonstrate adaptability by capitalising on these human-altered landscapes (Berger-Tal and Saltz 2019). We found seven species in all three habitat types. However, six species were found in only one habitat; five of which (*P. margaritophorus*, *D. caudolineatus*, *R. chrysargos, A. muelleri* and *X. trianguligerus*) were detected only in national park forest which is also the most diverse habitat on the island.

The data underscores the importance of conservation initiatives. Given the finite nature of an island, as habitat degradation escalates, the available space for species diminishes, intensifying competition for resources.

## Discussion

Our study documented 21 snake species representing 20 genera on Ko Pha-ngan, significantly augmenting the four previous records maintained by the Department of National Parks, Wildlife and Plant Conservation (2018) with 17 newly-recorded species. Forest associated species recorded exclusively within National Park Forest included *P. margaritophorus*, *D. caudolineatus*, *R. chrysargos*, *A. muelleri* and *X. trianguligerus* (Table 1), emphasising the importance of intact forest habitats for several specialised taxa. The presence of threatened species, such as *P. korros* (near threatened) and *O. bungarus* (vulnerable), alongside the island endemic *Oligodon phangan*, further emphasises the importance of baseline field inventories and the island's unique conservation value.

National park forest (NPF) hosts the highest snake diversity, supported by both the Shannon–Wiener Index (2.61) and Simpson’s Diversity Index (0.898), consistent with its less disturbed condition, although human settlements exhibited slightly higher richness. Collectively, these results suggest that intact forest continues to suport the most diverse snake assemblage on Ko Phan-gan, while modified habitats remain important for several widespread generalist species.

Edge habitats in HS and HDF support adaptable species like *A. prasina* and *Lycodon capucinus*, which appear tolerant of human dominated landscapes, while more specialised species, such as *P. margaritophorus*, *D. caudolineatus* and *R. chrysargos* remain restricted to forested regions. The detection of fossorial species (underground dwelling), such as *C. jodiae* across all habitats, likely reflects detection bias, as human activities such as digging, construction and gardening may expose otherwise cryptic species.

Ko Pha-ngan's present day snake assemblage is strongly influenced by its geological history. As part of the Sibumasa Terrane and the Southeast Asian tin belt, the island formed part of exposed Sunda Shelf during periods of lowered sea level, when terrestrial dispersal between the island and mainland Southeast Asia was possible (Sathiamurthy and Voris 2006). Consequently, much of the contemporary fauna is composed of widespread continental species that likely colonised the island prior to holocene isolation. Rather than producing a highly distinct snake assemblage, subsequent isolation appears to have influenced overall species richness while retaining a strong continental faunal signature.

Regional island comparisons provide context for evaluating whether the richness observed on Ko Pha-ngan is unusually high or low relative to islands differing in area, isolation and habitat condition. As an example, Phuket, the largest island in Thailand (543 km^2^), lies much closer to the mainland and harbours approximately approximately 60 snake species (Pauwels et al. 2002, Uetz et al. 2023, Visoot et al. 2023, Wilson 2024), 18 of which also occur on Ko Pha-ngan illustrating how isolation and area interact to structure island assemblages. The high proportion of shared species indicates that Ko Pha-ngan retains a strong Sunda Shelf continental fauna inherited through historical mainland connectivity (MacArthur and Wilson 1967, Dawson et al. 2016); however, the substantially lower species richness relative to Phuket is consistent with the effects of island area and long-term isolation on species persistence and colonisation.

The influence of island size and historical connectivity becomes clearer when Ko Pha-ngan is compared with other tropical islands in the region. Tarutao and the Phi Phi Archipelago report lower snake diversity (Lerdrungroj and Taksintum 2018), suggesting that Ko Pha-ngan supports a relatively high richness despite its size (125 km²). Bidong Island, the largest island at 1 km^2^ in the Archipelago of the same name, located approximately 14 km from the mainland coast of Peninsular Malaysia in the South China Sea, documents only three snake species (*L. capucinus*, *M. reticulatus*, *I. braminus*) (Fatihah-Syafiq et al. 2020, Abdul Rahman et al. 2022). Ko Man, approximately 7 km from the mainland in the Gulf of Thailand, supports five species, three of which (*I. braminus*, *M. reticulatus* and *C. ornata*) also occur on Ko Pha-ngan (Chan-ard and Makchai 2011), providing another regional example of how area, habitat availability and land-use history interact to influence diversity.

Habitat loss and fragmentation remain the primary conservation threats on Ko Pha-ngan. Globally, reptiles are increasingly threatened by habitat destruction and climate change (Böhm et al. 2013, Hanson and McElroy 2015). On islands, effective restoration should retain structural microhabitats such as fallen logs, course woody debris and leaf litter, which provides essential refugia for reptiles and amphibians (Kanowski et al. 2006). Replacement of native forest with monoculture plantations may, therefore, reduce both prey availability and habitat suitability for forest-dependent snakes.

The processes currently affecting Ko Pha-ngan are not unique. Studies of island ecosystems elswhere have demonstrated that rapid urban expansion, habitat fragmentation and inefficient land use reduce ecological integrity, while sustainable planning and forest restoration substantially improve ecosystem resilience (Chi et al. 2020). These broader patterns suggest that the trajectory observed on Ko Pha-ngan reflects challenges faced by many rapidly developing islands throughout Asia.

The detection of *O. bungarus* and *P. korros* highlights the need for targeted conservation strategies. These species, along with restricted-range endemics like *O. phangan*, face increasing risks from habitat degradation and fragmentation. Protecting the island’s National Park Forest remains critical, as it provides a refuge for forest-dependent species and maintains ecological stability.

Community reported rescue records documented frequent snake encounters within human settlements during the study period, suggesting that public education campaigns could help reduce unnecessary perrsecution and improve co-existence (National Research Council 1992), particularly in human settlements where adaptable species like *Naja kaouthia* are frequently encountered.

## Supplementary Material

XML Treatment for Ahaetulla prasina

XML Treatment for Boiga cyanea

XML Treatment for Chrysopelea ornata

XML Treatment for Coelognathus radiatus

XML Treatment for Dendrelaphis caudolineatus

XML Treatment for Lycodon capucinus

XML Treatment for Lycodon davisonii

XML Treatment for Oligodon phangan

XML Treatment for Ptyas korros

XML Treatment for Rhabdophis chrysargos

XML Treatment for Xenochrophis triangulagaris

XML Treatment for Cylindrophis jodiae

XML Treatment for Calliophis maculiceps

XML Treatment for Naja kaouthia

XML Treatment for Ophiophagus bungarus

XML Treatment for Homalopsis buccata

XML Treatment for Pareas margaritophorus

XML Treatment for Malayopython reticulatus

XML Treatment for Indotyphlops braminus

XML Treatment for Argyrophis muelleri

XML Treatment for Xenopeltis unicolor

## Figures and Tables

**Table 1.** Snake species list and detection by habitat of Ko Pha-ngan, Surat Thani Province, Thailand. LC = Least Concern, NT = Near Threatened, VU = Vulnerable, HS = Human Settlement, HDF = Human Disturbed Forest, NPF = National Park Forest, Opp. = opportunistic, Trap = obtained through a live trap, Rescue = obtained through a rescue. *New record from the Than Sadet - Ko Pha-ngan National Park checklist. ^+^ Endemic

Family	Species	IUCN Status	Total		Habitat Type			Detection method				
				HS		HDF	NPF	Survey	Opp.	Trap		
Pareidae	Pareas margaritophorus*	LC	3	0		0	3	0	1		2	
Cylindrophiidae	Cylindrophis jodiae*	LC	8	4		3	1	3	4		1	
Colubridae	Ahaetulla prasina*	LC	52	8		32	12	45	7		0	
	Boiga cyanea*	LC	18	1		7	10	17	0		1	
	Chrysopelea ornata*	LC	8	3		2	3	0	8		0	
	*Coelognathus radiatus*	LC	5	0		3	2	0	3		2	
	Dendrelaphis caudolineatus*	LC	1	0		0	1	1	0		0	
	Lycodon capucinus*	LC	18	0		12	6	12	0		6	
	Lycodon davisonii*	LC	5	0		3	2	5	0		0	
	Oligodon phangan*+	LC	7	0		2	5	5	0		2	
	Ptyas korros*	NT	3	0		1	2	0	5		0	
	Rhabdophis chrysargos*	LC	2	0		0	2	2	0		0	
	Xenochrophis trianguligerus*	LC	1	0		0	1	1	0		0	
Homalopsidae	Homalopsis buccata*	LC	7	7		0	0	7	0		0	
Pythonidae	*Malayopython reticulatus*	LC	9	3		4	2	5	4		0	
Elapidae	Calliophis maculiceps*	LC	3	2		0	1	3	0		0	
	*Naja kaouthia*	LC	3	1		1	1	0	3		0	
	*Ophiophagus bungarus*	VU	3	0		1	2	0	3		0	
Typhlopidae	Indotyphlops braminus*	LC	3	2		0	1	2	1		0	
	Argyrophis muelleri*	LC	1	0		0	1	0	1		0	
Xenopeltidae	Xenopeltis unicolor*	LC	11	4		6	1	11	0		0	
Total			171	35		77	59	119	40		14	

**Table 2.** Diversity index in different habitat types with and without rescue data. HS = human settlement, HDF = human-disturbed forest, NPF = national park forest.

Index	HS	HDF	NPF
Species richness	11	13	20
Shannon–Wiener	2.10	1.96	2.61
Simpson’s Diversity Index (1 – λ)	0.859	0.779	0.898
